# Taking the Bite

**Published:** 1906-07

**Authors:** Stewart J. Spencer

**Affiliations:** Chattanooga, Tenn.


### TAKING THE BITE.

By STEWART J. SPENCER, Chattanooga, Tenn.

The articulating impression, or bite, is best taken in wax, although guttapercha, modelling compound and even plaster of Parise have been used for this operation. Pure fresh beeswax is rather too soft, and therefore too easily bent in removal from mouth, or insubsequent manipulation. The addition of paraffine supplies the resired hardness. Boiling, with the addition of a little sulphuric acid, also improves and toughens wax; at the same time rendering it aseptic.

There are three ways of taking the bitt; viz., the biting into a lump of soft waw; the "True-bite-qlate" method; and the base plate method. These we will refer to as No. 1, No. 2. and No. 3. In the last method (No. 3) a base plate, whether of metal, valcanite, or wax, is made from the model; which necessitates a second sitting; or, at least, the keeping the patient in the office until the model can be taken from the impression and the base plate taken from the model, or from dies. This method was used entirely in the olden days of gold and and silver plates, ere the advent of vulcanite; but when vulcanite came, then came the temptation to take the bite in the easy method No. 1; and then, later, came the Trus-bite-plate system, as a half-way between the other two; a little more work than the "mash bite" methdr of No. 1, but having the advantage that it could be done without detaining the patient or requiring another sitting, as necessitated by No. 3.

he great objection to the No. 1 bite method is that ifthe patient's mandible is thrown either forward or sideward from its normal closure, (as it is apt to be because of the effort required to bite into the lump of wax), there is no way of detecting the error until the teeth are tried in the mouth. This is true, however, chiefly of cases where both jaws are toothless.

On the contrary, if teeth remain in both jaws, and if any two of them occlude, this method answers very well indeed. because the worn surfaces of the teeth will indicate the correct occlusion, and if the bite has been taken wrong, correction may be made, by means of these occluding teeth, or the articulator.

For full dentures No. 1, is not to be recommended, because of its great liability to wrong bite. But if the dentists persists in using it, he should do so with mere precautions:

First find the desired amount of opening to be given the jaws. As a rule, the length of the nose is the distance that should be given from nose to chin; the eyes being situate in the middle of the face, measuring from summit of skull. Therefore by requesting the patient to open the mouth until these distances are seen to agree, as you observe the patient's profile, you may get the correct amount of opening. Trim down a strip of wood to the amount the jaws should open, and insert this strip in the lump of wax, telling patient to bite until his jaws touch it.

If this strip is not used the jaws are apt to close too far; which not only tend to greater protrusion of the maudible than would otherwise occur, but also, because wax is nearly bitten through, rendering easily bent out of shape while the model is being pressed into it (for the lower jaw will always in these very close bites protrude far beyond the upper); thus leaving the wax easily bent out of shape and also it will necessitate opening the bite on the articulator; the which, unless the wax bite has been positioned exactly as far forward from the print of the articulator as it was while in the mouth from the natural joint, the posterior teeth will be found to occlude earlier in the mouth than in the articulator.

When this No. 1 bite is used for full dentures, or for those where are no remaining anterior natural teeth to serve as guides in positioning the porcelian teeth, it ought to remove from the mouth, trimmed along the leuccal and labial surfaces and replaced. Then a few upper teeth should be placed in the wax until the dentist has determined their correct positions, firstly, with regard to the median line, secondly, with regard to their showing from under the lip, up or down; and thirdly, with regard to their position anterio-posterierly. There are no rigid rules for this, but ordinarily an upper central incisor should extend downward so that its edge is on the same line as the lip when the lips are closed, and when the lips are opened to the extent they are in ordinary speech so that two-thirds or even more of said incisor should appear. With very long or very short lips, however, this rule must be modified; with the long lip, the incisor showing less, and with the short lip, more.

It is not always possible to get the porcelain teeth, when thus making trial of position, to fit exactly to place anterio-posteriorly, especially if they are section teeth, without grinding.

Many dentists fall into the error of giving too much protrusion to section anterior teeth.

These who prefer not to do this trial opposition work at this stage, but to run the risk of having to shift all the teeth at a later stage, should make marks on the wax to indicate the median line and the line of the lips (either when closed or approximately when speaking), transfer these to the model when it has been placed in the wax bite.

To be sure that the model fits fully down into the was bite, the dentist may cut away sections of wax from the buccal and labial walls in three places until level with the ridge. It can then readily be seen whether or not the model goes home.

Various machines have been mad and method recommended for preventing a misclosure of the jaws in taking the bite. None of theseare, I think, infallible. Those which aim at forcibly preventing protrusion by passing a machine, or cord, or even a napkin, around from the chin to the back of the neck are open to the serious objection that they may force the manuible back beyond its normal position in occlusion. The only correct way to obtain the desired result seems to be by repeated closures of the patient's jaws. Even then, it is doubtful, for it is wonderful how persicently some patients will bite wrong,—either by closing constantly in a false position or by closing in so many different positions as to leave a doubt that any one on which we may pick is correct. I think the safest and best method is to induce them, if possible, to make the masticating bite, as in eating. This is very likely to result in correct final occlusion.

For such repeated closures, and especially for thismasticatory bite method, the True-bite plates present themselves. There are a number of these plates, but we refer only to the pair intended for use when one or both jaws are edentulous. With them the patient bites into warm wax, as with method No. 1, but the plates divide the wax into two pieces, and thus permit of repeated clousers after the final. The amount of opening of the bite should be determined as before described, and two bite pieces of soft wood should be cut so that their united thickness, together with that of the plates, equals the des[illegible] opening; There should then be waxed to the plates, to prevent the jaws from closing farther than is desired. The wax is now placed on the plates and if lower teeth remain it is better to not insert both plates at once, but insert the lower first, pressing it to correct position. After both are inserted and closure made, they should be removed from the mouth and trimmed of all surplus wax, (as this tend to displace the impressions during the further operations), then replaced, and the correct bite gotten by repeated clousers, as above described. Scratch both upper and lower wax and notice by this scratch if the plates comes to the same place or each bite. If in doubt that the patient is persistently biting wrong, have him thrust up chin.

The previous remarks about trying in teeth, and about the medain line of lip, etc. apply here.

The No. 3 of taking the bite is best done by first making the baseplate of metal or vulcanite. If of wax only, strengthen it by thin iron bar.

The baseplates being made, a roll of wax is attached to aech coated with soapstone and then these are placed in the mouth and roughly brought together, then removed and separated, and their occlusal surfaces trimmed with a knife till they occlude correctly, being frequently placed in the mouth to determine when this is. The division line ought to be on the same plane as that intended for the teeth; which can now, (a few of them at least) be placed in their positions as before described. Not only the position of the teeth, but the amount of rubber for plumpers, labial and buccal, should now be determined; the wax should be cut from or build up on these trial plates until the proper fullness of cheek and of lip, especially that where the loss of the cuspid have left a sunken place near the wings of the nose, has been determined. This wax is not removed, as in No. 1 and No. 2.

When lower teeth remain, the upper wax should be trimmed to occlude with them, then their correct position marked by a scratch in front and one at the side, then a strip of soft, warmed wax spread over the occlusal surface of the upper wax and the lower teeth made slightly to indent same, the operator observing by hisscratches that the bite is correctly given. Then an impression of the lower teeth must be taken, a plaster cast gotten from copid impression, and the occluding surfaces of its teeth placed in the above mentioned indentions of the upper wax, and these be waxed together for placing on articulating frame.

It will be observed that for cases where a full upper or lower denture is t boe made while natural teeth remain in the opposite jaw, this last method is much more lengthy then that with the True-bite plates.

Where a partial plate, or a bridge, is intended to supply teeth on only one side of the mouth, it is usually best to take an impression of the entire upper and lower dentures, instead of one of that side only. The impression trays made to take both impression and bite at one time are therefore contra-indicated; the more so because they tend to a wrong occlusion, —the patient frequently thrusting his pair out sideways on the side where such impression is being taken. The reasons for taking a full bite will appear in our article on setting up the teeth.

(Continued next month.)

